# Bioleaching: urban mining option to curb the menace of E-waste challenge

**DOI:** 10.1080/21655979.2020.1775988

**Published:** 2020-06-14

**Authors:** Shashi Arya, Sunil Kumar

**Affiliations:** a Technology Development Centre (TDC), CSIR-National Environmental Engineering Research Institute (CSIR-NEERI), Nagpur, India; b Technology Development Centre (TDC), Academy of Scientific and Innovative Research (AcSIR), Ghaziabad, India

**Keywords:** E-waste, printed circuit board, informal recycling, hydrometallurgy, pyro-metallurgy, bioleaching

## Abstract

Resource Recovery from Waste Electronics has emerged as one of the most imperative processes due to its pressing challenges all over the world. The Printed Circuit Board (PCB) is one of the typical E-waste components that comprise large varieties of metals and nonmetals. Urban Mining of these metals has received major attention all over the world. The existing treatment procedures used extensively for the resource extraction are hydrometallurgy and pyro-metallurgy and crude recycling practices in the informal sector. However, these methods are prone to cause secondary pollutants with certain drawbacks. Also, the existing informal recycling procedures resulted in insignificant occupational health hazards and severe environmental threats. The application of biotechnology is extensively exploited for metal extraction and emerged as one of the sustainable and eco-friendly tools. However, a limited field-scale study is prevailing in the realm of resource recovery from E-waste using bioleaching method. Hence, the application of bioleaching requires more attention and technical know-how in developing countries to curtail crude practices. The application of bioleaching in E-waste, including its available methods, kinetics mechanism associated opportunities, and barriers, have been discussed in this paper. A glance of E-waste management in India and the menace of 95% crude E-waste recycling are also elaborated. The incentives toward profit, socio-economic, and environmentally sustainable approaches have been delineated based on critical analysis of the available literature.

## Graphical Abstract

**Figure:**
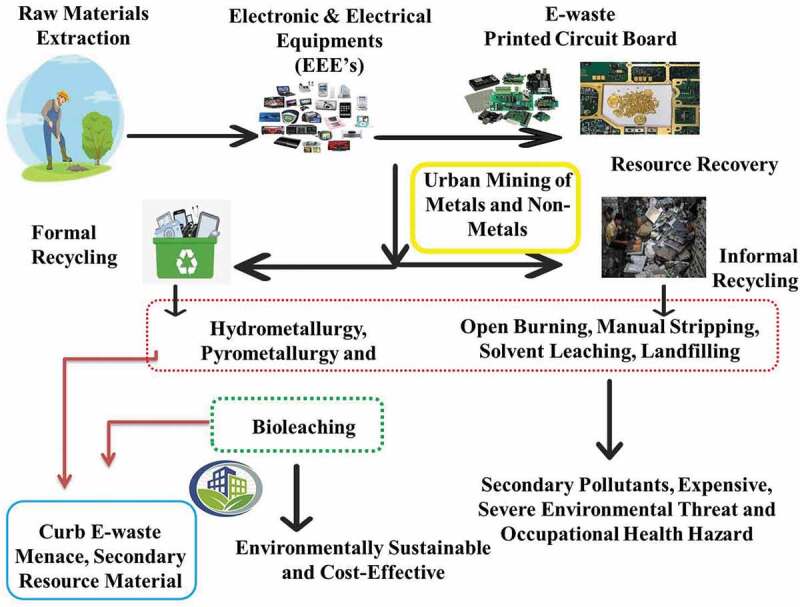

## 1. Introduction

The continuous advancement in the Information and Communication Technology (ICT) and the transformation of Digital Society has impelled enormous Electrical and Electronic Equipment’s (EEE’s) all over the world. The transformation to digital society is also evident from the article by Mckinsey and Company “Manufacturing’s next act 2015 [1]. Similarly, a Digital Flagship Programme 2015 [2] in India, launched by Prime Minister of India, led toward creating its own EEE aimed to foster the skill and employment opportunities. This rapid and enormous transformation is leading at a high rate production of new and advanced appliances every day, replacing the older gadgets resulting in massive obsolete products into the bins termed as ‘Waste Electrical and Electronic Equipment’s (WEEE) or E-waste.’ The E-waste generation has become one of the quickest developing waste streams on the planet exceeding three times the production of the Municipal Solid Waste (MSW) [3,4]. Among different categories of E-waste classified, the number of white goods, such as computer and mobile waste,is huge in numbers [5]. For example, India is one of the second largest producers of mobile waste all over the world nearly four times that generated in the USA [6]. The annual global E-waste generation varies between 20 and 50 Million tons (Mt) in parallel to the population growth and consumer demand and is predicted to increase 3–5% annually [7]. Presently, the global per capita E-waste generation is 6.1 kg/person over 50 Mt of E-waste,*i.e*., equivalent to the weight of all existing aircraft made until now [8]. Of the total E-waste, only 20% is collected for resource recovery, and the rest 80% (35.8 Mt) is left unreported and abandoned [9]. Based on the observations and trends of the global E-waste generation growth rate, it is expected to rise to 52.2 Mt by 2025 [9]. China is the leading country among many developed countries (stringent legislation and infrastructure toward the E-waste management) and developing countries (large amounts of E-waste are dumped illegally, the practice of informal recycling and haphazard management of the waste) [10,11]for producing 7.2 Mt of E-waste trailed by 6.3 Mt in the USA, 2.1 Mt inJapan, 2.0 Mt in India, and 1.9 Mt in Germany [10–12]. E-waste is a potential hub for secondary resource generation due to valuable and precious metals, such as iron, steel, aluminum, barium, copper, bismuth, nickel, etc. and palladium, silver, and gold and become a core for urban mining [13,14]. However, it is considered in the hazardous waste stream due to the presence of heavy metals, such as lead, cadmium, arsenic, antimony, beryllium, chromium, and mercury also plastics with Brominated Flame Retardants (Chlorine, bromine, nitrogen or phosphorus) Polychlorobiphenyl and Polyvinyl chloride [7]. When the E-waste components are exposed for resource recovery or subjected under a procedure, the element gets liberated into the environment, exceeding the permissible limit, and becomes a potential threat to the human health and environment [15–17]. The European Union (EU) and other developed countries have tended to the issue of the E-waste by taking crucial and immediate action plans. Also, most of the countries embraced the best exploratory & sustainable procedures and techniques for resource recovery from E-waste. However, few countries, namely, India, China, Pakistan, Sri Lanka, Bhutan, Nepal, Ghana, Cambodia, etc. are facing substantial deficits towards the sustainable treatment of E-waste. A number of the patterns in utilization and generation procedures are unsustainable and pose a real threat to the environment and human wellbeing. The factors, such as lack of infrastructure, no stringent implementation of E-waste rules and legislation, and crippled framework of EoL (End of Life) products, the entire E-waste value chain are hindered in India and many other developing nations [18]. Therefore, the ideal and effective utilization of distinctive assets, improvement in cleaner items, and minimization of waste is a portion of the issues that need to be addressed. At the same time, guaranteeing the financial development and upgrading the entire E-waste value-chain in the country is realized to be improved.

Since E-waste is one of the secondary resources for many metals and nonmetals, it is recognized as a potential source for extended business opportunities. As estimated, the global E-waste generation is highly in demand when subjected to appropriate treatment options enables toward the economic growth of the country and reduces the need for mining virgin raw materials. However, it’s very saddening that only 15% of the E-waste is only subjected for valuable extraction [19]. Hence, unlike the primary resources that requires deep mining and complicated processing; this secondary resource is readily available on the surface of the mother earth and can be easily extracted by ‘Urban Mining’ through an eco-efficient recycling procedure, *i.e*., Bioleaching.

### 1.1. Urban mining

**Figure 1.**
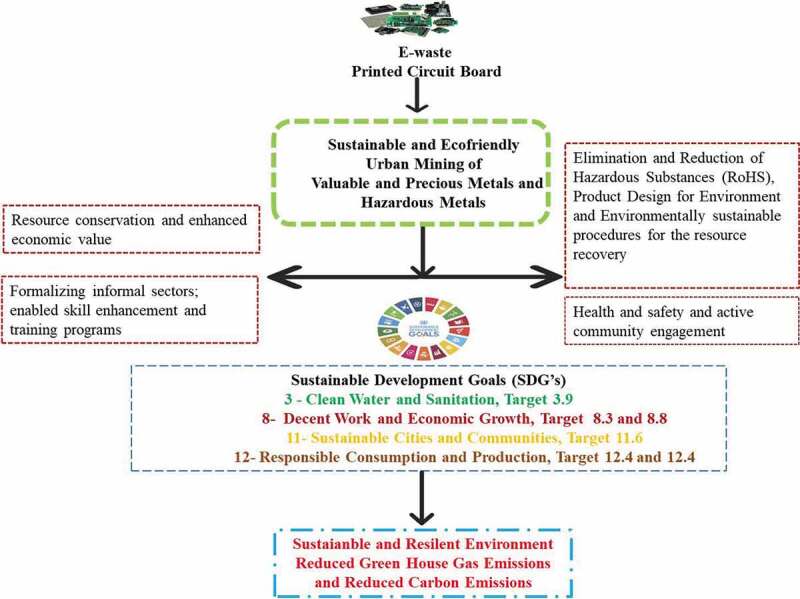
Urban mining and its scope toward SDG.

‘Urban mining is the concept and an approach for up-keeping the environment and ensuring the advancement of resource conservation through 3 R (Reuse, Recycling, and Recovery) of valuable and precious materials from waste’ [20]. Among various categories of waste, E-waste is considered as the heart for urban mining. It represents a stock and potential hub for metals, plastics, glass, and others that can be possibly retrieved after the EoL of the product. According to the global E-waste Monitor report, the global E-waste generated in 2016, *i.e*., 44.7 Mt, consists of approximately €55 billion worth of precious and other valuable metals [9,21]. Urban mining is extensively exploited in China and is highly appreciated for providing alternative solutions to meet the carrying capacity of the country’s resources in manufacturing the EEE at large [22]. According to the US Geological Survey, 2018, China is individually accounted for approximately 81% of the total global production of Rare Earth Elements (REE) [23]. Also, as reported by UNU, Germany, 2018, Europe individually can mine 2 Mt of EoLbatteries/year (90% of the batteries are lead-based, and remaining 10% are nickel-metal hydride, zinc, and lithium-based) that represents a rich source of secondary raw materials (CRM’s) approximately 78,000 tonnes of lithium, 21,000 tonnes of cobalt and 114,000 tonnes of manganese [24]. According to the study reports, effective urban mining has the potential toward saving the waste material worth around 21 USD billion [25]. The Prospecting Secondary Raw Materials in the Urban Mine and Mining Wastes (ProSUM) project in Europe has reported to harmonized and endeavored the urban mining concept and achieved a successful and dynamic development. A study conducted by UNU, 2018 reported that approximately 40 various critical metals can be retrieved from a single smartphone of which individually gold is accounted for its 25–30 times purity over the primary gold ore. As reported by the United Nations General Assembly in New York in 2015 [26], E-waste management is directly associated with the accomplishment of the Sustainable Development Goals (SDG’s) 2030 Agenda. Hence, in the assembly, it was urged by all countries to maintain a sustainable approach toward E-waste management with a focus on reducing the waste generation. The strategic implementation of urban mining of E-waste leads toward maximizing the resource conservation and increasing the economic value, planning and designing the infrastructure of cities, positive approach toward the health & safety and employment opportunities as well-reduced greenhouse gases and ultimately contributing toward achieving the (SDG’s) 2030 and its targets shown in Figure 1.

The specified targets that linked to the E-waste management are mentioned hereunder [26].

**SDG Target 3.9**: By 2030, substantially reduce the number of deaths and illnesses from hazardous chemicals and air, water, and soil pollution and contamination;

**SDG Target 8.3**: Promote development-oriented policies that support productive activities, decent job creation, entrepreneurship, creativity and innovation, and encourage the formalization and growth of micro-, small- and medium-sized enterprises, including through access to financial services;

**SDG Target 8.8**: Protect labor rights and promote safe and secure working environments for all workers, including migrant workers, in particular women migrants, and those in precarious employment;

**SDG Target 11.6**: By 2030, reduce the adverse per capita environmental impact of cities, including by paying particular attention to air quality and municipal and other waste management;

**SDG Target 12.4**: By 2020, achieve the environmentally sound management of chemicals and all wastes throughout their life cycle, in accordance with agreed international frameworks, and significantly reduce their release to air, water and soil in order to minimize their adverse impacts on human health and the environment;

**SDG Target 12.5**: By 2030, substantially reduce waste generation through prevention, reduction, repair, recycling and reuse. Mining E-waste has envisaged toward reduced Co_2_emission approximately less than 80% per unit of gold to that of primary excavation and process.

Despite tremendous advancement and research explored toward resource recovery from E-waste, still many countries are at the verge in handling the perils of E-waste. This paper is focused to get an outlook that India and many other developing countries have an immense potential to curb the E-waste hazards through implementing an integrated sustainable and cost-effective approach. Hence, considering the colossal menace of the E-waste generation all over the world and the existing treatment options, it has critically become mandatory to curb the threat of the E-waste in an eco-friendly way and further scaling up the **Bioleaching Technology**. Also, a better understanding of the process, technical know how and additional suggestions are required for its implementation in developing countries. Based on the above context, extensive literature has been reviewed as a title search in the ‘Bioleaching: an effective approach for E-waste recycling’ from different sources. Reports published by the several agencies, such as Global E-waste Monitor, Toxic Link, India, ASSOCHAM, 2018, SteP Initiative, were also explored for the existing norms and regulations framed toward controlling the menace of the E-waste. Secondary information regarding the current treatment options has been collected from the recyclers and company profiles.

### 1.2. Waste printed circuit boards (WPCBs)

Almost all the entire E-devices are snuggled with PCB implanted in complicated structure and design ensures the smooth functioning and conductivity of the gadget [27].PCB represents approximately 3–6% of the total weight of E-waste components and 40% of precious and valuable metals and nonmetals embedded all over the boards [28–30]. Thus, the PCB’s have gained much attention and considered as one of the significant hotspots for resource recovery and extended business opportunities for value generation [31,32]. However, the critical amalgamation of the toxic metals along with the others made them a huge concern all over the world. PCBs are generally of two types *i.e*., FR-4 and FR-2. FR-4 PCB forms the multilayer structure coated with a copper layer on fiberglass embedded through epoxy resin. These sorts are commonly utilized in small devices whereas FR-2 is made of a single layer coated with copper on a cellulose fibers board or glass layer, utilized enormously in large household appliances. A typical composition of PCB comprises 0.3% to 0.4%of precious elements (20–500 ppm gold, 200–3000 ppm silver, and 10–200 ppm platinum), 28% of valuable elements (copper 10–20%, nickel 1–3%, aluminum and steel) and toxic metals, such as lead, cadmium, arsenic and antimony including 49% of glass & ceramic and 19% plastics [33]. These metals are coupled intact with one another and form complicated structure and design [34,35]. As estimated, one metric ton of PCB contains 80 to 1500 kg of gold and 160–210 kg of copper [36,37]. PCBs are additionally accessible in various sizes and shapes varying from less than 1–2 cm ± 10 cm in different hues, such as blue, green, black and red depending on the type and size of the gadget [38]. Hence, the knowhow of the PCB’s is much required to invest in the resource recovery business including its treatment methods. Though various methods are being exploited for the resource recovery from PCB’s, however, significant and sustainable measures and approach are limited.

## 2. Existing E-waste resource recovery options

**Figure 2.**
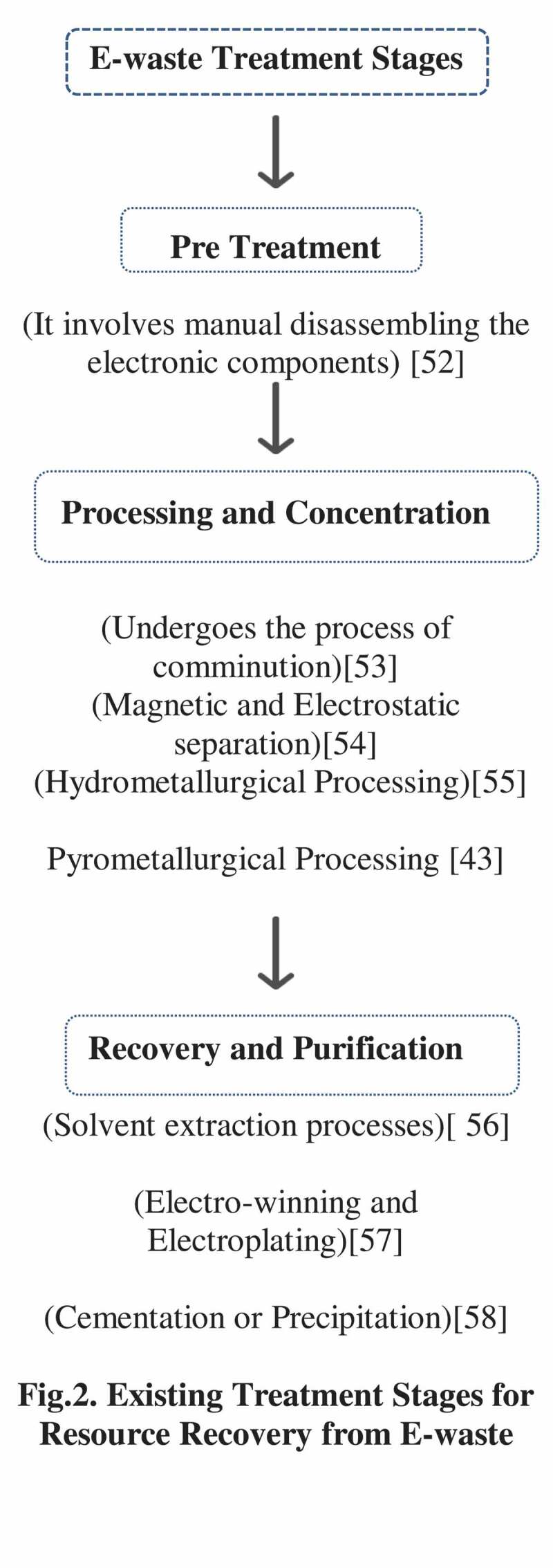
Existing treatment stages for resource recovery from E-waste.

Resource recovery from PCB’s is of great economic interest and a potential hotspot for urban mining of valuable and precious metals, such as copper, gold, silver, nickel, palladium and platinum, iron. It is also highly appreciable due to its advanced degree of purity over metals extracted from ores. Approximately 34 kg of gold can be retrieved by recycling one million cell phones [39]. The resource recovery from E-waste is initially obtained by various traditional methods, such as collection, segregation, pretreatment (dismantling, hammering, chiseling), processing and purification presented in Figure 2. The various methods adopted for the processing, and extraction of the metals are the combination of the either physico-chemical or individual. The processes, such as hydrometallurgy *viz*., acid bath, and chemical leaching [40], pyrometallurgy *viz.*, roasting, pyrolysis, burning, heating, smelting [41–43], and electrometallurgical [44–46] are discussed. Also, approximate cost estimation for resource recovery from E-waste and its potential market value has been presented in Tables 1 and 2. Indeed, the methods are used extensively toward resource recovery and contribute in reducing the E-waste menace. At the same time, the methods are assumed to be highly complex requiring consumption of huge volumes of acidic and basic solvents, higher temperatures leading to be expensive as well asenergy intensive [47,48]. Also, the generation of secondary pollutants, such as dust particles, toxic gases (dioxin and furans), acid leachates leads to water pollution and acid water drainage has become a huge challenge due to its potential threat to human health and environmental parameters [49–51].

**Table 1.** Investment in resource recovery including its treatment method [Adapted and modified from Priya and Hait, 2017 [15], Chatterjee, 2012 [59] and CAMA, 2006 [60]].

		Approximate Investment US$/kg of E-waste	
S. No.	Treatment method	Hydrometallurgy	Pyrometallurgy
1	E-waste collection	5.199	5.199
2	Manpower charges for pretreatment (dismantling and segregation)	1.039	1.039
3	Processing and concentration (shredding etc.)	0.208	0.208
4	Smelting process	1.549	^a^NA
5	Recovery and purification (by wet chemical leaching)	^a^NA	1.243

^a^NA 1 USD = INR 60

**Table 2.** Typical market value of the resource recovery from E-waste [Adapted and modified from Toxic Link, 2018 [83] and [60–64]].

E-Waste Material	Resource Recovery	Market Value INR	US $
CRT’s	Copper, Plastics, Glass, Lead, Aluminum	Copper: 421.60/kg Aluminum: 132.10/kg	5.48/kg 1.72/kg
Liquid Crystal Display (LCD)	Indium, tin and Copper	Iron Material: 40–50/kg	0.52–0.65/kg
PCB	Tin, Copper, Lead, Iron, Bromine, Nickel	Lead: 140.50/kg Indium: 25,000–45,000/kg	1.83/kg 325.22–585.40/kg
Mobile Phones	Copper, Silver, Gold, Palladium, Cobalt	Silver: 1400–1500/kg Gold: 3,283/gm Palladium: 3,033/gm Plastic Scrap: 20–65/kg Transparent Glass: 100–150/square feet	18.21–19.51/kg 42.71/gm 39.46/gm 0.26–0.85/kg 1.30–1.95/square feet

Resource Recovery based on the process treatment

INR and US $ as per 2019 Market Price

### 2.1. Hydrometallurgy

Hydrometallurgy also known as chemical leaching process involves dissolving the waste in appropriate solvents or acids or ammoniacal solutions [65], such as concentrated nitric acids (HNO_3_) [66], hydrochloric acid (HCl), sulfuric acid (H_2_SO_4_) on industrial scale, aqua regia and few alkalis followed by pretreatment of the waste [27]. The process of hydrometallurgy involves the electrochemical etching of the metals and is attained through different techniques and combinations of chemical leaching, such as cyanide, halide, Thiourea and Thiosuphlate. It is also possibly achieved by mixing the chemicals with ligands, such as Ethylenediaminetetraacetic acid (EDTA), Diethylene Triamine Pentacetate (DTPA), Nitrilotriacetate (NTA) and Oxalate as a chelating agent. Among all, EDTA has the potential to recover approximately 10% lead, from E-waste also chromium, copper and zinc [67,68]. This method has been considered as a more selective route for resource recovery particularly copper from PCB’s as it is present in the elemental form [27].

The process is considered to consume a high amount of energy [69,70] and requires high operational cost [71] with severe threat to the environment and human health. It also requires the additional expenses toward the post treatment and management of acidic wastewater generated in bulk amounts [72,73].

### 2.2. Pyro-metallurgy

The process involves the separation of precious and base metals from inerts material embedded in WEEE. To ensure its efficiency, high-temperature melting furnaces are used in conjunction with special melting fondants. Few leading companies, such as Umicore, Dowa, X-Strata and Boliden are practicing pyrometallurgy for resource recovery [71]. Umicore is extensively recycling about 17 metals including 7 precious metals [16]. It involves the process of heating the waste sample in an inert atmosphere at a temperature range of 400–700 °C [27]. After certain interval of time the components, such as wood, rubber, paper and plastics start decomposing and gets converted into volatile substances that can be readily used in the chemical industry or can be collected in the form oils or gases as a substitute for energy generation or high-value chemical products [74]. The PCB’s of waste computer have estimated to generate 22.7% oil that can be used as a raw material in chemical industries, 4.7% gases (having high calorific value) and 70% of copper-rich residue [75]. However, the pyrolysis of E-waste component prone to cause severe health and environmental problems due to the release of toxic gases like dioxin and furan [72,76]. Sintering is also one of the processes that involves the compaction and formation of a solid mass of waste sample or material by heat and pressure without melting it to the point of liquefaction. The process is generally applicable to a condition where the mineral deposits are used with metals, ceramics, plastics and other materials [77].

**Figure 3.**
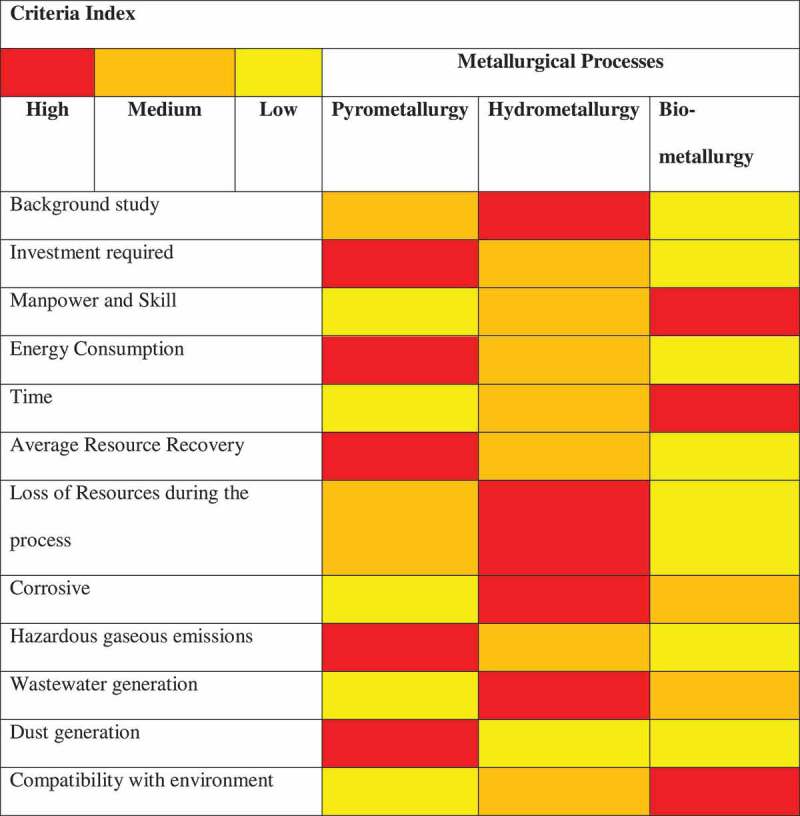
Comparative assessment of treatment options for resource recovery from E-waste [Adapted and modified from Priya and Hait, 2017 [15]].

However, the conventional methods of treatment are deliberated to be environmentally unsustainable, highly expensive, requires vast infrastructure including maintenance cost of the equipment’s and energy consumption. The comparative assessment of the treatment methods and criteria index adopted for resource recovery emphasizing its advantages and disadvantages has been shown in Figure 3.

### 2.3. Informal recycling

As estimated, approximately 1% *i.e*., 15 million people of total population living in the urban areas in most of developing countries are engaged in the informal sector [78]. In most of the developing countries, a huge amount of E-waste ends up in the informal sector under crude practices. In this case, most of the E-waste disposal and recycling methods are unsafe and does not come under the ambit of the government regulations. Environment and human health concerns related to operations in the informal sector are causing illegal extraction of precious metals and posing a severe threat with the release of toxic gases (dioxin, furans, CO, sulfur, lead, cadmium, hexavalent chromium VI) leading to air pollution and deteriorated health conditions of the people engaged in the profession as well as the common man [79]. And the E-waste that is left abandoned and not recycled contributes to 4.25% of the greenhouse gas emissions [80]. Therefore, an extensive approach is required to identify the loopholes in the informal E-waste management and treatment scenario with an attempt to integrate informal collectors into the formal sector and adopt the best E-waste take-back scheme suitable for developing countries.

## 3. Occupational health hazard and environmental threat associated with existing treatment methods of E-waste

An occupational hazard is associated with the occupational risk to the person/workers which may have a serious potential impact on the health of the person. Similarly, occupational health hazard defines the harm that has occurred to the worker in the profession and that has taken the form of illness or risks to life. According to the section 1 of **Occupational Health & Safety (OHAS**), the ‘Occupational illness is a situation resulted due to continuous acquaintance in a typical occupation or profession subjected by any means of agent *i.e*., physically, chemically or biologically to an extreme that the standard metabolic functional mechanisms gets affected, and the condition of the person starts deteriorating’ [81]. The potential sources and associated risk related to the occupational health hazard in E-waste is unauthorized collection, inappropriate segregation, dismantling and recycling processes without any skills and training and disassembly of the products its associated components that might have certain resell value.

The people involved in the occupation are exposed to various diseases, such as nausea, vomiting, reduced immune systems due to deposition of arsenic and other heavy metals in the blood vessels; fragile bones, lung damage, and kidney diseases due to cadmium, asthma, skin ulcers, severe redness; and swelling of skin due to chromium, brain and kidney damage may be death, pregnant women exposed to lead may cause miscarriage due to lead. Assumed routes of entry of metals led to hazard presented in the section.

Routes of entry

There are three primary routes of entry of toxic material into the body.

- Ingestion – Most of the time, the workers engaged in this profession inadvertently get exposed to toxic chemicals through the routes of mouth or swallowed (while consuming any eatables). Most of the emissions that are soluble prone to be more dangerous as they get absorbed in the intestine and can cause the internal damaging of the organs.
- Absorption – Since most of the activities during handling the E-waste is done without any prior protection equipment’s and mask. Also, most of the segregation, dismantling, and mechanical separation are done with bare hands the skin gets in contact with the toxic chemicals while processing the E-waste, most of the chemicals get absorbed in the skin and then into the blood system. As reported, most of the E-waste recyclers have reported suffering from skin diseases, such as reddening and itching of the skin.
- Inhalation – Most of the time while processing the E-waste, the toxic gases are emitted and inhaled in the form of vapors, gases, mists or particulates and get deposited in the respiratory tract as a result, the airborne contaminant can be easily absorbed through tissue. A personal interview was conducted by the first author on 12 May 2019 in Mumbai Manish Market a biggest electronic gadget, and repair in whole sale reported to inhale a chemical typically called as ‘FLUX’ every time the nozzle is opened.

The burning of E-waste components, such as CRT, cable wires, plastics, PCB’s printed wiring boards (PWB’s) etc. emits toxic gases, such as furans and dioxin a colorless, flammable, highly volatile liquid and carcinogenic [82]. These burned trashes create dangerous toxic smoke, consists of many tiny particles. People exposed to smoke are also believed to be at increased risk of pneumonia and other infectious lung diseases. Elders, children’s, pregnant women’s and people with heart or respiratory diseases are most at risk from breathing toxic smoke and smoke particles [83]. Similarly, dumping and landfilling of E-wastes can lead to leaching of lead, cadmium, arsenic and many other toxic elements into the groundwater. As published by Prasad and Vithanage 2019 [84] around 600 m^3^ of water is contaminated with cadmium by recycling the battery of a single mobile phone. For extraction of gold and silver, the recyclers often use an acid bath to dissolve the waste material & other metals and their remaining parts are washed and dumped directly into the nearby waterbodies. With the span of the time due to chemical reaction and certain changes in the landfills, the toxic elements start leaching contaminating the surface and groundwater pollution along with exacerbating the high levels of heavy metals in the nearby areas and surrounding land. The workers in rural areas are unaware of the impact of the contamination of drinking water and surface water with heavy metals.

In purview of the above illustrated critical scenario, various associations, Government bodies, Environment Protection Ministries, Pollution Control Boards, Urban Local Bodies, Municipal Corporations Research and Development Institutions, NGO’s are working immensely to curb the menace of E-waste. Few of them are UNU Germany, ProSUM Project and Solving the E-waste Problem (StEP) Initiative have observed the menace of the E-waste and are constantly involved to curtail the perils of the hazards of the E-waste. StEP initiative has developed a concept called Best -of-2-Worlds (Bo2 W) that is focused to merge the technical as well as logical aspects for the advanced global best practices and processing facilities for E-waste. Also, a StEP initiative delineated that dismantling is one of the highly efficient and economically viable options to recover metals from E-waste. However, the components, such as PCB and the batteries waste would demand high tech technologies for resource recovery [24,85,86].

## 4. Bioleaching

Resource recovery from mineral ores using oxidizing bacteria is historically ancient civilizations in Asia [87]. Based on the sustainable and ecofriendly ability of a micro-organism to transform the wastes into a valuable resource, bioleaching is considered as one of the most promising processes over the last ten centuries [88]. Thus, the microbial leaching has emerged as an important asset and renders as economically sound with the reduced environmental problem than conventional methods. Traditional method of E-waste treatment hassles foremost energy and reagent expenses to create metal concentrates, coupled with laborious work, such as size reduction, segregation and metal extraction. However, it is understood that the E-waste leads to toxic emissions and contribute environmental footprint from CO_2_ emissions thereby contaminating air quality, groundwater and soil quality. Therefore, the bioleaching method has desired to be sustainable technique with lower energy costs and improved environmental legacies [47]. It also offers a great opportunity for employment as well as the reduced burden on virgin resources. Bioleaching of E-waste has gain much attention due to its technologically advancement in terms of sustainability and as intermediate technologies especially in developed nations. Bioleaching uses microorganisms to facilitate leaching of minerals and offers significant economic benefits over traditional metallurgy due to reduced infrastructure costs, and low energy input through operation at ambient pressure and temperature [89,90]. Although bioleaching is used commercially to process copper, zinc, nickel and cobalt ores, there is no commercial exploitation of bio-leaching of E-waste streams on the large-scale despite the excellent potential for preferential leaching of metals, such as gold and other precious metals [28]. Few microbes listed extensively for the resource recovery from PCB are *Acidithiobacillus ferroxidans (*mesophilic-chemolithoautotrophic bacteria) [91,92]. *Sulphobacillusthermo sulfidooxidans and Thermoplasma acidophilum (*acidophilic moderately thermophilic bacteria) [93,94] and *Chromobacterium violaceum*an

**Figure 4.**
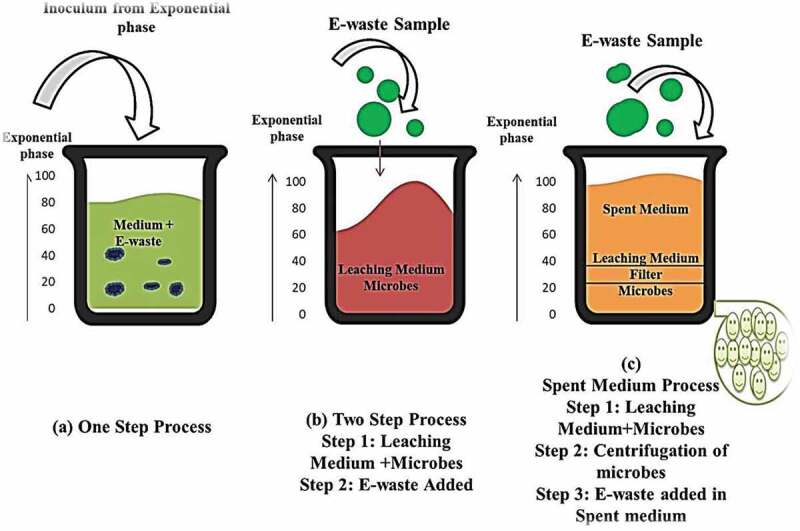
Variations and different steps of bioleaching of E-waste.

Bioleaching works on the principle of the ability of the metals to get dissolved by the oxidizing microorganisms [95]. According to Breed and Hansford in 1999 [94], studies on the mechanisms and kinetics of bioleaching, states ‘that the process involves three critical sub-process *i.e*., sulphide mineral, bacteria and high redox potential’. The mechanism of the bioleaching process is elucidated by the estimation of O_2_ and CO_2_ consumed proportions coupled with the estimation of redox potentials and it also assist the kinetics of the sub-processes involved during the leaching [95]. The process of the bioleaching is evident by two strong controlling sub-process *i.e*., chemical leaching of the pyrite by ferric iron and the other process is the ferrous iron product is oxidized to ferric ion under the influence of the microbes. The kinetic study is applied to determine the ratio between the ferrous to ferric [96] under optimal conditions, such as pH, temperature, pulp density, etc. The initial incidence of the microorganisms (acidophilic autotrophic iron and sulfur oxidizing bacteria) used for metal solubilization was identified by Colmer and Hinkle from Acid Mine Drainage (AMD) [97]. Bioleaching is basically accompanied by two different mechanisms of the interaction of the microorganism over metals*i.e*., direct bacterial leaching and indirect bacterial leaching. The dissolution of the mineral is basically accompanied by one or several mechanisms, such as complexolysis, acidolysis and redoxolysis [98] depending on the type of the microorganism involved in the leaching process. Based on the favorable conditions the microorganism has to get adapted to the waste material to trigger the leaching efficiency. Thus, this tendency of the microorganism and the waste materials has been explored in various steps that includes the bioleaching in the contact of the microbes with the samples, such as one-step and two-step bioleaching and bioleaching wherein there is no contact between the microbes and the sample known as spent medium bioleaching [99–101] as shown in Figure 4.

Post bioleaching process the extraction of metals is a crucial and complex procedure. Due to no. of metals and nonmetals embedded over the waste material, a multicomponent solution is obtained. Therefore, the extraction of target metals is a complicated process. Various methods have been explored, such as electrowinning, electroplating and solvent extraction process from the leached solvents. According to Gotfryd and Pietek, 2013 [102] and Panada et al., 2012 [103] solvent extraction is one of the effective method for recovery of the desired metal from the solution. The experiment conducted by Willner et al., 2014 [104], reported the extraction of copper by the solvent extraction method using organic solvent – LIX 860 N-IC of an optimal concentration of about 5%. The rate of copper extracted was between 97% and 98.5% and the iron ranged between 25% and 3.8%.

### 4.2. Direct bacterial leaching

In this process, the microorganism binds with the base of the waste and oxidizes the element followed by several enzymatically catalyzed steps as given in Equations (1), (2) and (3) [95].

(1) $$4FeS_{2}+14O_{2}+4H_{2}O^{bacteria}\to 4FeSO_{4}+4H_{2}SO_{4}$$

(2) $$4FeSO4+O_{2}+2H_{2}S{O_{4}}^{bacteria}\to 2Fe_{2}{\left(SO_{4}\right)}_{3}+2H_{2}O$$

Also, direct bacterial leaching can be described as per the reaction given in Equation 3.

(3) $$MeS+2{O_{2}}^{bacteria}\to MeSO_{4}$$

#### 4.2.1. One-step bioleaching process

Generally, one step leaching process is the basic step that engages the whole bioleaching mechanism in one step *i.e*., the microorganism (the mother culture) is grown in a flask in a aseptic condition and once the population count of the cells reaches to maximum *i.e*., exponential phase, the same is taken for the bioleaching as inoculum. Further, the inoculum is added into another flask that consists of the nutrient medium including E-waste sample [105]. The whole process is performed in an aseptic condition to avoid any contaminations and left for certain time period for the generation of ferric iron that gradually solubilizes the copper from insoluble Cu^0^ form to soluble form Cu^2+^using iron as an energy source (Equations 4–5) [106]. Similarly, the mechanism of bioleaching of metals, such as Zn, Ni and Al would solubilize depending on their thermodynamics reactions (Equations 6–8)[106]. Due to complicated structure and design the amalgamation of elements especially the heavy metals when liberated during the process inhibits the growth of the microorganisms thus the rate and the growth of the reaction and population count gets declined, respectively. Hence, one step bioleaching process assumes to have certain difficulties.

(4) $$4Fe^{2+}+{O_{2}}^{Fe-oxidizers}\to 4Fe^{3+}+H_{2}O$$

(5) $$2Fe^{3+}+Cu^{0}2Fe^{2+}+Cu^{2+}\Delta G^{0}=-82.9kL/mol$$

(6) $$2Fe^{3+}+Zn^{0}2Fe^{2+}+Zn^{2+}\Delta G^{0}=-295.4kL/mol$$

(7) $$2Fe^{3+}+Ni^{0}2Fe^{2+}+Ni^{2+}\Delta G^{0}=-196.6kL/mol$$

(8) $$3Fe^{3+}+Al^{0}3Fe^{2+}+Al^{3+}\Delta G^{0}=-1085.2kL/mol$$

#### 4.2.2. Two-step bioleaching process

Direct growth of micro-organisms along with E-waste in the medium is not preferred due to the toxic nature of the waste [91]. Hence, two-step bioleaching processes is desired to enhance the growth of the microorganisms without any hindrance or toxicity effect. In this condition, the desired microbe is cultured in a respective nutrient medium under required conditions [105,107]. Once the population count of the microbes reaches to the exponential phase, the waste sample is added into the microbial cultured medium as a second step and subjected for the leaching process. In this step, the inhibition of the organism’s growth is reduced and creates optimum conditions for the microbial growth. This process is more appropriate [108] and attractive as the rate of reaction and metal solubilization is faster as well as higher even at the conditions of maximum pulp density [109].

### 4.3. Indirect bacterial leaching

In Indirect bacterial leaching, the microorganisms oxidize the waste by producing a lixiviant and solubilize the metal without being in direct contact with the base of the waste as given in Equations (9) and (10).

(9) $$MeS+Fe_{2}{\left(SO_{4}\right)}_{3}\to MeFeSO_{4}+2FeSO_{4}+S^{0}$$

(10) $$2S^{0}+3O_{2}+2H_{2}O^{bacteria}\to 2H_{2}SO_{4}$$

#### 4.3.1. Spent medium process

In this case, initially, the two-step bioleaching process is adopted for the microbial growth and along with lixiviants. Further, the microbial culture is separated and the collected spent medium is mixed with the E-waste sample [47,48,109]. This process is highly recommended for gold recovery as compared to other process and also it was envisaged to reduce the toxicity of the PCB on the growth of the microorganisms that was a major limitation in the one-step process. A study conducted by Wu et al. 2018 [111] at State Key Laboratory of Bioreactor Engineering in East China University reported that the bioleaching of valuable metals from PCB by using a supernatant of a microbial culture of oxidizing bacteria has the potential to recover 93.4% of copper as whole from 100 g/L PCB concentrates in only 9 days. Cyanogenic microorganisms are preferable in this process due to its ability to produce HCN. In case of two-step bioleaching process HCN gets reduced that leads to low gold extraction.

### 4.4. Factors influencing E-waste bioleaching

To understand the technical knowhow toward maximum resource recovery, the evaluation of the potential influential factors*i.e*., Physical (temperature, particle size and pulp density, waste component and metallic composition), Chemical (pH, culture medium, solid/liquid ratio) and Biological (microbial strain, microbial interactions, growth medium) in E-waste bioleaching is critically important [112].

An ideal temperature for any microorganism is a highly influential parameter. As investigated so far, an ideal temperature for the growth of microorganism and their metabolic activity range widely between 25°C and 75°C. The average temperatures for microbial growth involved in bioleaching of E-waste are mostly mesophilic, moderate thermophilic and thermophilic. According to Indian climatic conditions and seasonal variation [113], the temperature lies between 0°C and 45°C varying every 3–4 months in different states that absolutely fits the ambient conditions for the mesophilic and moderate thermophilic microbial growth and feasible at lab-scale conditions thus reducing the expenses toward maintenance of the temperature for a field-scale study application [Author’s Perspective]. According to the bioleaching process can be obtained at a range of temperatures, i.e.,, from ambient to 80°C to a demonstration plant [114]. Pulp density is defined as the amount of addition of various metals and plastic consisting of crushed fine powder of PCBs to the selective species inoculums [115]. Pulp density has direct control over the growth of the species that trigger the rate of leaching and solubilize the metal. Optimum pulp density ranges between 10 and 15 mg/l. Pulp density is directly proportional to the extraction of metals *i.e*., higher the pulp density, higher the chances of the metal recovery. However, at a certain level, the pulp density has assumed to have inhibitory effects on the microbial population count, such as in one step leaching process, the elevated pulp density inhibits the growth of the microbes due to release of heavy metals and ultimately reducing the leaching performance, slow agitation and limiting the mass-transfer [116]. Hence, the non-contact bioleachinglike spent medium is preferred to overcome with certain issues. Therefore, having depth knowledge (of the optimum relationship between the pulp density and target metal recovery) is quiet essential in designing a bioreactor at industrial-scale and make sure it is profitable in terms of maximum yield and economically viable [47]. In addition to the pulp density, the waste quantity, size and metal dosage concentration also plays a significant role in the optimum growth of the microorganisms and its efficiency. At the same time, the ratio of the both solid (the waste material) and the liquid (the growth media that acts as energy sources for microorganisms) should have a balanced combination to govern the bioleaching process. Experiment conducted by Zhu et al., 2011 [117]recommended that the small particle size waste is better for metal extraction; however, according to Adhapure et al. [118] large size particle of waste material is more preferred to avoid the formation of precipitate contamination problem additionally simplifying the overall metal recovery shown in Table 3.

**Table 3.** Microorganism and conditions for bioleaching.

Waste Component	Microorganisms	Temp (^o^C)	pH	Stirring Time (RPM)	Solid/Liquid Ratio (%w/v)	Time Duration (Days)	Metal Extracted (%)	Authors
WEEE Shredding dust	*Acidithiobacillus ferroxidans*	30	1.0–3.5	150	0.5–2	8	Ce, Eu, Ne-99 Lan, Y-80	[116]
	*Pseudomonas putida*	30	-	150	1	30 hours	Au: 48	
WEEE	*Acidithiobacillus ferroxidans + Acidithiobacillus thioxidans*	30	2.1–0.9	150	0.5–1	21	Al, Cu, Ni, Zn- 90	[91]
PCBs	*Sulphobacillus Thermosulfidooxidans+acidophilic isolate*	45	2.0	180	1	18	Cu: 89 Al: 79 Ni: 81 Zn: 83	[93]
PCBs	*Acidithiobacillus ferroxidans + Acidithiobacillus thioxidans*	Ambient	1.0–1.6	150	1	7	Cu:98	[124]
TV PCBs	*Acidithiobacillus ferroxidans + Leptosperillum ferroxidans +Acidithiobacillus thioxidans*	35	1.7	175	1	4.8	Cu:89	[125]
PCBs	Mixed Acidophilic bacteria	30	2.0	160	1.2	1.8	Cu: 96.8 Al: 88.2 Zn: 91.6	[117]
PC PCBs	*Acidithiobacillus ferroxidans*	30	3.0	170	2	0.8	Cu: 100 Ni: 100	[126]
PC PCBs	*Acidithiobacillus ferroxidans*	30	2.2	170	1.5	3	Al: 75.4 Cu: 96.8 Zn: 83.8	[127]
CRT Fluorescent Powder	*Acidithiobacillus ferroxidans + Leptosperillum ferroxidans +Acidithiobacillus thioxidans*	30	1.7	175	10	16	Y: 70	[127]
PCBs	*Acidithiobacillus ferroxidans*	30	2.0	165	2.5	4	Cu: 100	[128]
PCBs (4–10 mm)	*Acidithiobacillus ferroxidans*	30	1.8–2.5	170	1	28	Cu: 94.8	[129]
PCBs (1 mm)	*Acidithiobacillus ferroxidans*	30	2.0	160	10(v/v)	7	Cu 32.4	[143]
PCB Powder	*Leptospiriuulm ferrriphilum and Sulfobacillus thermosulfidooxidans* (Supernatant)	55	1.2	200	-	2 hours	Cu: 100	[111]

**pH** is one of the most important factors that enhance the growth of the micro-organisms and their ability to solubilize the metals from the waste material. Based on various studies and research [119], microorganisms’ ranges from 1.0 to 4.0 pH are supposed to give high-efficiency metal solubilization. Since, microorganisms have varied optimum growth pH, therefore, it is highly recommended toward the selection of the appropriate microorganisms also based on the nature of the target metal [120]. Resource recovery intervened by iron and sulfur oxidizers [28] including fungi require acidic pH whereas Cyanogenic bacteria are effectively active in alkaline pH above 9.0. It is also critically important to monitor and regulate optimum pH conditions for microbial cultures toward the production of lixivant agents that are crucial for the metal leaching from the waste. For example, *Aspergillus niger* produces different organic acids [121] at different pH, such as citric acid is produced at low pH values (≤2) while pH values (≥4) it produces gluconic and oxalic acid [109].

Despite all the above factors, the nutrient for the growth of the bacteria assumes a very important and significant role for the enhanced population count of the microbes. Among the broader range of microorganisms, the most commonly used microorganisms for bioleaching are acidophilus and chemolithotrophic microbial consortia of*Acidithiobacillus ferroxidans, Letospririllum ferroxidans and other* heterotrophic microbes, such as species of *Sulphobacillus* [106]. The Gram-negative bacillus bacterium oxidizes Fe^2+^ to derive energy and solubilizes the metals. Hence, iron and sulfur compounds coupled with ammonium, phosphate and magnesium salts are supplemented for optimum growth conditions. One of the most common nutrient medium for microorganism used follows the Silverman and Lundgren known as 9 K medium that comprises,3.0 g (NH_4_)2SO_4_, 0.1 g KCl, 0.5 g MgSO_4_•7H_2_O, 0.5 g K_2_HPO_4_, 0.01 g Ca(NO_3_)2, and 74.5 g FeSO_4_•7H_2_O in 1 L distilled water [122]. Despite of its most promising technologies among all other exiting methods for resource extractions from E-waste [123], bioleaching has certain limitations, such as toxicity of Waste PCB for microbial growth and handling of the microbial cultures. Also, the method is only limited to the laboratory scale set-up and hence, appropriate estimation of the investment is still at the infancy stage. Also, the technology is assumed to tackle a small quantity of E-waste sample due to the viability of the microorganisms for a longer period of time.

#### 4.4.1. Significance of bioleaching over chemical leaching

**Figure 5.**
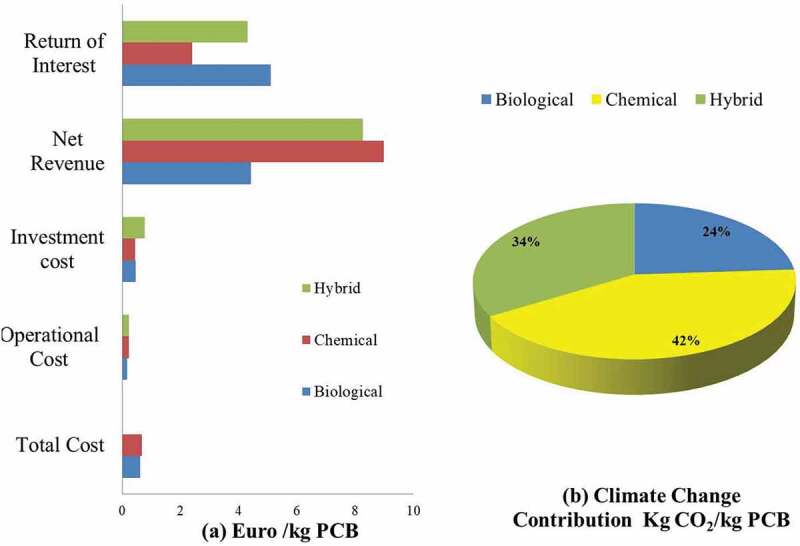
Techno-economic and environmental sustainability evaluation of existing methods of resource recovery from waste PCBs [131]. (a) Comparative capital expenditure and revenue generation of different methods of E-waste resource recovery. (b) Influence of the respective technology on climate change and the contribution of carbon dioxide per PCB [92].

According to Pant et al., 2012 [130], usually chemical leaching leads to higher resource leaching whereas in the case of bioleaching, the yield is quiet low. However, several researches have been reported that bioleaching has shown significant yield of metals over chemical leaching. For example, in a spent medium leaching the FCC catalysts using *G. Oxydans* with 10–15 mM gluconic acid has been more effective. This indicates that the supernatant of microbial culture contributes toward higher yield of the metals leaching. As reported by Isildar [131], the biological extraction of metals is techno-economically more effective as well as environmentally sustainable shown in Figure 5. in terms of managing the E-waste menace along with enhanced resource recovery than hydrometallurgy, pyrometallurgy, open burning and crude & open acid leaching practices.

## 5. E-waste management in India -at a glance

India being one of the most emerging and largest developing countries with the second largest populated country in the world has reported to generate 2.1 Mt of E-waste in 2019 [132]. A study conducted by ASSOCHAM-KPMG, 2018 has reported that India is one of the world’s second-largest mobile markets [133] and likewise the fourth largest producer of E-waste [8].Additionally, the illegal trading and importing of scarp waste from other countries is one of the mammoth tasks to handle the waste management at a large [134,135]. Also, due to the unavailability of appropriate treatment methods, India is currently facing a huge threat toward the entire value chain management of the waste along with the booming challenge of 95% backyard recycling [136–139]. Hence, the remaining 5% of the E-waste is outsourced by the formal recyclers in association with few IT companies, such as Tata Consultancy Services (TCS), WIPRO, HP, Infosys and many others at the cost of 0.13–0.20 USD/kg (INR. 10–15/kg). The processed E-waste at the plant is further sold at the price near around 0.10–0.13 USD/kg (INR. 8–10/kg) for shredded plastics, 0.18–0.29 USD/kg (INR. 14–22/kg) for scrap metallic parts, 0.91–1.04 USD/kg (INR. 70–80/kg) for alunimium parts and 0.013–0.26 USD/kg (INR. 1–2/kg) for Cathode Ray Tube (CRT) glass to the traders or manufactures. However, the marginal profit in the business is reported to be nearly 25–30%; hence, the capacity for enhanced effective management and treatment is yet to be explored and delineated [140].Therefore, the entire E-waste value chain *i.e*., collection, storage, transportation, treatment or recycling, the process are yet to be well regulated. As estimated approximately 70% of the E-waste in India is computer waste followed by 13% telecommunication equipment, 8% electrical equipment and 7% medical equipment [141]. The PCB of a computer waste comprises with around 30.1% of metal content and 14.6% of copper [142]. Hence, such a forecast indicates the imperative need to cope with the opportunities and challenges associated with E-waste in India. Taking into the account and the menace of the E-waste overflow in most of the regions in India, the rules and regulations were drafted as E-waste (Management & Handling) Rules in 2011. Further, new modifications have been made in 2016 followed by 2018 by The Ministry of Environment Forestry and Climate Change (MoEf&CC) with special emphasis on the strengthening of the rules and extending the roles of the producers, manufacturers and consumers of electronic goods and including the components of E-waste management. The primary objectives of the rules are to channelize the E-waste treatment through best practices, such as sustainable and eco-friendly treatment methods, circular resource management, Polluter’s Pays Principle (PPP), Extended Producer Responsibility (EPR) and creating awareness among the people for their active participation. Public participation is quiet an essential factor in maintaining the cradle to grave E-waste value chain of any city or town. However, most of the NGO’s such as Toxic Link, Center for Science and Environment (CSE) and Chintan and Environment Action Research Program and ULB’s are working in collaboration with the public to develop systematic plan and layout for effective management and sustainable communities, yet the progress is slow and much attention is required on an immediate basis. Seelampur an E-waste market in New Delhi, India is one of the largest E-scrap in the country where a large of no. of different categories of waste, such as personal computers, PCBs, mobile phones, televisions, refrigerators, printers, other household electrical gadgets are collected and compiled from different states involving local rag pickers, scrap dealers [83]. Most of the valuable components mainly copper, gold silver, nickel is extracted from these scraps by around 30,000 localpeople (children age 12), women’s, and men’s, in this trade. However, as reported the most of the populations engaged in this profession work independently and only a few works in collaboration with big traders over a marginal profit of around INR 200/$2 per day. Hence, considering the huge generation of E-waste, it became imperative to curb the E-waste menace in a safe and organized mode with sufficient resources and environmentally sound technologies. Since, a large portion of the waste is recycled informally in India most of the resources are lost or stolen and the heavy metals exposed to crude recycling deteriorate the health and environmental quality. Also, as reported in the articles and existing recycling companies in India, bioleaching of E-waste is limited and is at an infant stage [28]. Hence, much attention is required at an emergent basis and therefore, bioleaching can be extensively exploited for maximum resource recovery from computer waste as it contains a higher amount of copper, gold and silver [143]. Thus, Bioleaching of E-waste especially from computer waste could be possibly open wide opportunities for maximum resource recovery extending the opportunities for economic growth when delineated appropriately and explored at the filed scale.

## 6. Conclusion and recommendations

Environmental concerns and the need for resource recycling have made the E-waste processing an economically viable option and are required to make this process more efficient and economic. Significantly, bioleaching has boomed most of the recyclers to recover valuable and precious metals from E-waste. Hence, bioleaching presents as an advanced technology over pyrometallurgy and hydrometallurgy. It also aims to be beneficial to industries, small units engaged in waste management activities, Central and State Pollution Control Boards besides providing jobs to skilled and semi-skilled educational youth. The resources recovered from E-waste can be used as a circular economy approach by the industries thereby reducing the burden of raw material for manufacturing other products. Resource recovery using bioleaching technology has remarkable benefits on economic growth, public health and sustainable environment. It has gained much attention due to its simpler and cost-effective approach, reduced energy requirement, and negligible contaminations. However, the process is constrained to have certain limitations, such as slow& minimum yielding outputs, space requirement and proper infrastructure and current inadequacy for treating the complicated complex mixture of metals. The technology is associated with two broad aspects of sustainability-related to the environment, resource & health protection and profitability related to the value & utilization of recovered products. However, based on the existing scenario in the developing countries, there is a requirement of decentralized approach on a priority basis for improvement in E-waste management from cradle to grave. Also, advancement in the technologies and overcoming the limitation in the existing treatment options for more sustainable resilient environment, health, safety and resource conservation is also very much needed.

### Authors’ perspective

Despite rapid advancement in the technologies and treatment procedures, each of the method has its own challenges and opportunities. Also, due to the critical and complexity of the resources embedded in the E-waste, none of the procedure can be exploited individually. Hence, to curb the E-waste menace, firstly an entire E-waste value chain is needed to be explored to delineate a sustainable model with a focus toward an integrated approach. The resource recovery from E-waste using bioleaching method has been studied extensively and explored at a laboratory scale. However, the field-scale study is limited and at an infancy stage in India and other developing nations. For an instance, in India, the E-waste collection system is still very poor due to no. of reasons, such as data privacy, emotional attachments, etc. Also, like MSW collections systems, there is no separate collection of E-waste items. However, few initiatives have been taken by the NGO’s as E-waste collection drive programs. Also, India is struggling with 95% of the E-waste in the informal sector and only 5% of the E-waste goes under the formal unit. India and many other developing countries are having a large scope in the field-scale implementation of the bioleaching method due to the availability of the E-waste material in a bulk amount. However, this is only possible with a sustainable and integrated model approach. The study is being conducted by the first author (a Ph. D. Scholar) addressing the above issues.
